# Exploring the recuperative potential of brassinosteroids and nano-biochar on growth, physiology, and yield of wheat under drought stress

**DOI:** 10.1038/s41598-023-42007-2

**Published:** 2023-09-11

**Authors:** Muhammad Aown Sammar Raza, Muhammad Arif Ibrahim, Allah Ditta, Rashid Iqbal, Muhammad Usman Aslam, Faqeer Muhammad, Shehzad Ali, Fatih Çiğ, Baber Ali, Rao Muhammad Ikram, Muhammad Noor Muzamil, Muhammed Habib ur Rahman, Mona S. Alwahibi, Mohamed S. Elshikh

**Affiliations:** 1 Department of Agronomy, Faculty of Agriculture and Environment, The Islamia University of Bahawalpur, Bahawalpur, 63100 Pakistan; 2 Department of Environmental Sciences, Shaheed Benazir Bhutto University Sheringal Dir (U), Sheringal, KPK Pakistan; 3 School of Biological Sciences, the University of Western Australia, Perth, WA 6009 Australia; 4 Department of Environmental Sciences, Quaid-i-Azam University, Islamabad, 45320 Pakistan; 5 Department of Field Crops, Faculty of Agriculture, Siirt University, Siirt, Turkey; 6 Department of Plant Sciences, Quaid-i-Azam University, Islamabad, 45320 Pakistan; 7 Department of Agronomy, MNS-University of Agriculture, Multan, Pakistan; 8 Institute of Crop Science and Resource Conservation (INRES), University of Bonn, Bonn, Germany; 9 Department of Seed Science and Technology, Institute of Plant Breeding and Biotechnology (IPBB), MNS-University of Agriculture, Multan, Pakistan; 10 Department of Botany and Microbiology, College of Science, King Saud University, Riyadh, 11451 Saudi Arabia

**Keywords:** Biotechnology, Plant sciences, Environmental sciences, Chemistry

## Abstract

Drought stress as a result of rapidly changing climatic conditions has a direct negative impact on crop production especially wheat which is the 2nd staple food crop. To fulfill the nutritional demand under rapidly declining water resources, there is a dire need to adopt a precise, and efficient approach in the form of different amendments. In this regard, the present study investigated the impact of nano-biochar (NBC) and brassinosteroids (BR) in enhancing the growth and productivity of wheat under different drought stress conditions. The field study comprised different combinations of amendments (control, NBC, BR, and NBC + BR) under three irrigation levels (D_0_, D_1_ and D_2_). Among different treatments, the synergistic approach (NBC + BR) resulted in the maximum increase in different growth and yield parameters under normal as well as drought stress conditions. With synergistic approach (NBC + BR), the maximum plant height (71.7 cm), spike length (17.1), number of fertile tillers m^–2^ (410), no. of spikelets spike^–1^ (19.1), no. of grains spike^–1^ (37.9), 1000 grain weight (37 g), grain yield (4079 kg ha^–1^), biological yield (10,502 kg ha^–1^), harvest index (43.5). In the case of physiological parameters such as leaf area index, relative water contents, chlorophyll contents, and stomatal conductance were maximally improved with the combined application of NBC and BR. The same treatment caused an increase of 54, 10, and 7% in N, P, and K contents in grains, respectively compared to the control treatment. Similarly, the antioxidant response was enhanced in wheat plants under drought stress with the combined application of NBC and BR. In conclusion, the combined application of NBC and BR caused a significant increase in the growth, physiological and yield attributes of wheat under drought stress.

## Introduction

Wheat growth and productivity are hampered due to stress possessed by drought conditions in arid and semiarid areas^1,2^. A negative effect can be seen in the plant’s photosynthetic machinery, especially in stomatal conductance, thylakoid electron transport, Calvin cycle, and CO_2_ assimilation^3–6^. Drought stress also disturbs the balance of the production of reactive oxygen species and antioxidant production system which causes the production and accumulation of ROS which ultimately leads to disruption and disorganization of cell membrane lipids and DNA strands^7–11^.

While plants have developed protective mechanisms including physiological, biochemical, and morphological against water scarcity^3,12^ during their evolution process e.g. enhancing signaling pathways of phytohormones in response to abiotic stress^13–17^. The production of brassinosteroids (BR) which are polyhydroxylated steroidal hormones and play many physiological and morphogenesis processes starting from seed germination to flowering and senescence of plants^18^. Moreover, abiotic stress is also controlled via BR application^19^ like (1) enhancing the activity of antioxidative enzymes^20^ ultimately reducing the production of superoxide anion^21^ (2) abscisic acid accumulation is reduced by (BR) application^22^ although this abscisic acid causes the closure of stomata under drought stress^20,23^ and (3) osmotic permeability of roots are being increased for more water uptake.

Nano-biochar (NBC) also mitigates the negative effect of drought stress on plant growth and yield e.g. sorghum, maize, and wheat^24–26^. This activity also helps in more water retention hence lower demand of the number of soil irrigation^27,28^ increasing the nutrient use efficiency^29,30^_,_ stimulating the activity of gibberellins and auxins and regulation of BR^31^, and enhancing the stomatal conductance, chlorophyll contents, cytotoxicity, and K^+^ contents in leaf^32^. This carbon-rich and cost-effective component is made through the process of pyrolysis of organic residues in the absence of oxygen^33,34^, resulting in highly porous and aromatic carbon contents^35^. It was worth seeing that this carbon-enriched compound stays longer in the soil as compared to many other organic residues such as compost hence, this mitigates and competes the climate change through carbon sequestration^36,37^. In upcoming years there was a dire need in growing crops worldwide to fulfill the food requirements of humans and animals^38^. Wheat holds a most important place in global food security^39–41^, which contributes nearly 40% towards total world food demand. During 2019, its global production was 757.4 million tons^42^.

Many high-yielding wheat cultivars have been introduced for increasing wheat productivity^43,44^, and these cultivars also uptake high amounts of mineral nutrients^45^, so appropriate techniques are also needed for the betterment of nutrient uptake to sustain the availability of limited resources. For example, the nitrogen use efficiency of wheat does not exceed 33% globally^42^, and other nutrient use efficiency does not exceed 50%. Under drought stress, these efficiencies also decrease considerably^46^ and this condition results in the decline in wheat productivity. Although many studies have investigated the sole positive effect of BR and NBC, the combined effect of both of these compounds has not yet been investigated so far.

Based on the above discussion, the present study hypothesized that the application of BR and NBC alone or in combination could enhance the growth, physiological, and yield attributes of wheat grown under different drought stress conditions. The objective of the present study was to investigate the impact of BR and NBC, alone or combined on the growth, physiological, biochemical, and yield attributes of wheat under different drought stress conditions.

## Materials and methods

### Soil analysis

Soil samples were taken from experimental plots through auger, before sowing, and placed in tagged polyethylene bags. These bags were shifted to Soil and Water Testing Laboratory, Regional Agriculture Research Institute Bahawalpur. Various physicochemical parameters were measured using standard methods. The soil sandy loam with pH = 7.22, electric conductivity = 2.54 dS m^–1^, organic matter = 0.90%, nitrogen = 1.57 mg g^–1^, available phosphorus = 6.63 mg kg^–1^ and available potassium = 115 mg kg^–1^. Weather measurement was noted after the experiment from the observatory unit which showed an average precipitation of 15.50 mm and a temperature of 28.17 °C during the growing season.

### Field experiment

A field experiment was conducted at the agronomic research area of UCA & ES, The Islamia University of Bahawalpur to study the effect of nano-biochar (NBC) and brassinosteroids (BR) on wheat under drought stress. The experiment was arranged as a randomized complete block design (RCBD) with the factorial arrangement, having four replications in 10 cm apart lines. Faisalabad 2008 cultivar, obtained from RARI (Regional Agriculture Research Institute Bahawalpur) was sown in a 15 m^2^ plot subjected to drought stress at tillering (D_1_) and drought stress at anthesis (D_2_) stages and the plots receiving normal irrigation were considered as control treatment (D_0_). Three treatments T_0_ = control, T_1_ = NBC (Nano-biochar) T_2_ = BR (Brassinosteroids), and T_3_ = NBC + BR (co-application of nano-biochar and BR) were applied to the plots. For Brassinosteroids treatment, 24-epibrassinolide (C_28_H_48_O_6_ MW = 480.7) was purchased from Sigma-Aldrich. Brassinosteroids (120 mg L^–1^) were applied twice (tillering and anthesis stages) through foliar spray while nano-biochar (0.75% w/w) was incorporated in the soil at the time of sowing. Nano-biochar was obtained from Shanghai Hainuo Carbon Industry Co., Ltd China. Three-acre inches per irrigation water was applied as per schedule except during respective tillering and anthesis stages of the treatments to induce the drought stress excluding the control plots. The control plots received four irrigations in total using the flood irrigation method. Tube well water with pH = 6.5 and EC = 886 µS cm^–1^ was used for irrigation purposes. Fertilizer was applied @ 120 kg N and 80 kg P_2_O_5_ per hectare, using urea and diammonium phosphate (DAP), respectively.

### Growth and yield parameters

Various yield and growth-related parameters were determined through the procedures discussed below. The number of fertile tillers was counted in per square meter from each plot. Fifteen plants were selected randomly from each treatment plot at the time of harvesting and their spike length and plant height were measured with measuring tape and then averaged. Spikes were then separated from each tiller to record the number of spikelets spike^–1^ and 1000 grain weight after manual threshing. At the time of harvesting, a manual method was used to cut the crop for reducing any loss. The harvested crop was tied in bundles and their biological yield was recorded with a weighing balance for each treatment.

### Leaf area index (LAI)

The total leaf area was measured by randomly selecting fifteen plants from every subplot and then the average was taken out. Hence, LAI was calculated by using the formula given by Watson^47^.

$${\text{LAI }} = {\text{ Leaf area}}/{\text{Land area}}.$$

### Harvest index (%)

It was calculated for each plot by using the following formula:

$$\mathrm{HI}=\frac{\mathrm{Economic\,\, yield }\;(\mathrm{grain\,\, yield})}{\mathrm{Biological\,\, yield }\;(\mathrm{grain}+\mathrm{straw})} \times 100.$$

### Physiological parameters

#### Leaf chlorophyll contents

Leaf chlorophyll contents were measured by using a UV/VIS spectrophotometer. Chlorophyll content was measured by using Arnon’s method^48^. Fresh leaves of 0.1 g were grounded and placed in 80% acetone overnight. After that sample was centrifuged for 5 min at 10,000 rpm. The absorbance was measured at 645 nm and 663 nm wavelength and chlorophyll was measured by the given formula:

$${\text{Chl a }} = \, \left[ {{12}.{7 }\left( {\text{OD 663}} \right) \, - {2}.{69 }\left( {\text{OD 645}} \right)} \right] \, \times {\text{ V}}/{1}000 \, \times {\text{ W}},$$

$${\text{Chl b }} = \, \left[ {{22}.{9 }\left( {\text{OD 645}} \right) - { 4}.{68 }\left( {\text{OD 663}} \right)} \right] \, \times {\text{ V}}/{1}000 \, \times {\text{ W}},$$

V is the supernatant volume and W is the fresh weight.

#### Relative water contents (%)

The third leaf from the top (fully expanded youngest leaf) of ten plants of each treatment was used to determine the leaf’s relative water content (RWC). Immediately after cutting at the base of the lamina, leaves were sealed within plastic bags and quickly transferred to the lab. Fresh weight (FW) was determined within 2 hours after the excision of leaves. Then turgid weight (TW) was obtained after soaking leaves in distilled water for 16–18 h at room temperature. After soaking, leaves were quickly and carefully blotted dry with tissue paper to calculate the turgid weight. Dry weight (DW) was obtained after oven during the leaf samples for 72 h at 70 °C. Relative water content was calculated by using the following formula^49^

$${\text{RWC }}\left( \% \right) \, = \, \left( {{\text{FW }}{-}{\text{ DW}}} \right)/ \, \left( {{\text{TW }}{-}{\text{ DW}}} \right) \, \times { 1}00,$$

where FW = fresh weight, DW = dry weight, TW = turgid weight.

#### Leaf stomatal conductance (mmol of H_2_O m^-2^ s^-1^)

Stomatal resistance/conductance measurements were made with an automatic porometer MK-3 (Delta-T Devices, Burwell Cambridge, England) Hertford, Herts, England).

### Grain quality parameters

NPK was measured for assessing the grain quality as per the method described by Wolf^50^.

### Antioxidant activities

Leaf (1 g) was ground in liquid nitrogen to get the enzyme extract. The obtained powder was added to 50 mM phosphate buffer (10 mL) at pH 7.0 and was then mixed with 1 mM ethylene diamine tetraacetic acid (EDTA) and 1% polyvinylpyrrolidone (PVP). The whole mixture was spun at 13,000 × g for 20 min at 4 °C. The resulting supernatant was used for the enzyme assay. H_2_O_2_ decomposition rate at 240 nm indicated the catalase (CAT) activity as proposed by Hwang et al.^51^. The CAT activity (U/mg protein) was estimated from the molar absorption coefficient of 40 mm^–1^ cm^–1^ for H_2_O_2_. Peroxidase (POD) activity was recorded as per the method given by Kar and Mishra^52^. The reaction mixture consisted of 10 μL of crude enzyme extract, 10 μL of 100 mM H_2_O_2_, 160 μL of 50 mM sodium acetate (pH 5.0), and 20 μL of 100 mM guaiacol. Absorbance was recorded at 450 nm. Superoxide dismutase (SOD) enzyme activity was observed through the measurement of 50% inhibition of the rate nitro blue tetrazolium chloride reduction^53^. The reaction mixture contained 130 mM methionine, 0.75 mM NBT, 0.05 M phosphate buffer (pH 7.0), 0.02 mM riboflavin, and 300 μL enzyme extract. The reaction mixture and blank were exposed to fluorescent light for 7 min and absorbance was taken at 560 nm.

### Statistical analysis

The collected data regarding various parameters were analyzed statistically through a two-way analysis of variance (ANOVA) using Statistix 8.1 software^53^. The difference among mean values was determined using the least significant difference (LSD) test at a 0.05 probability level. Microsoft Excel 2016 was used for the preparation of graphs and the calculation of means and standard error values.

### Ethics approval and consent to participate

The seeds variety (Faisalabad 2008 cultivar) was obtained from RARI (Regional Agriculture Research Institute), Bahawalpur, Pakistan. All the experiments were performed in accordance with relevant guidelines and regulations".

## Results

### Growth and yield attributes

Statistical analysis of data shows significant differences in plant height as the result of different treatments and drought stress levels (Table 1). Maximum plant height of 71.7 cm was recorded in D_0_ (Control) whereas statistically lowest plant height (54.99 cm) was obtained in D_2_ (Drought stress at anthesis stage). Treatment T_3_ (NBC + BR) resulted in the maximum plant height (71.7 cm) and it was 30.4% more in comparison to the control treatment. In relation to the interaction of both factors under study, a statistically significant (*p* ≤ 0.001) interaction was recorded (Table 2).

**Table 1:** Effect of nano-biochar (NBC) and brassinosteroids (BR) on plant height (cm), spike length (cm), no. of fertile tillers m^–2^, no. of spikelets spike^–1^, no. of grains spike^–1^, 1000 grain weight (g), grain yield (kg ha^–1^), biological yield (kg ha^–1^) and harvest index of wheat under drought stress.

	Control	NBC	BR	NBC + BR
Plant height (cm)				
Normal irrigation	63.8d*	66.7c	68.7b	71.7a
Drought stress at tillering stage	59.5e	58.6e	59.1e	59.1e
Drought stress at anthesis stage	54.9f	55.3f	55.8f	56.1f
Spike length (cm)				
Normal irrigation	14.1e	14.6 cd	15.5b	17.1a
Drought stress at tillering stage	11.2i	11.8 h	13.5f	14.3de
Drought stress at anthesis stage	12.4g	14.1e	14.8c	15.7b
No. of fertile tillers m^–2^				
Normal irrigation	402c	405b	410a	410a
Drought stress at tillering stage	310i	325g	342e	342e
Drought stress at anthesis stage	315h	323g	337f	345d
No. of spikelets spike^–1^				
Normal irrigation	16.7ef	18.6b	17.7c	19.1a
Drought stress at tillering stage	15.6hi	16.4f	16.8e	17.1d
Drought stress at anthesis stage	13.7j	15.5i	15.8h	16.0g
No. of grains spike^–1^				
Normal irrigation	33bc	34b	37a	38a
Drought stress at tillering stage	29e	31d	33bc	34b
Drought stress at anthesis stage	27f	29e	32cd	33bc
1000 grain weight (g)				
Normal irrigation	35.3b	35.8b	36.7a	37.0a
Drought stress at tillering stage	32.2d	32.8d	33.6c	33.7c
Drought stress at anthesis stage	25.5f	25.9f	27.0e	27.1e
Grain yield (kg ha^–1^)				
Normal irrigation	3707d	3854c	3976b	4079a
Drought stress at tillering stage	2949h	3196g	3309f	3353e
Drought stress at anthesis stage	2457l	2650k	2826j	2920i
Biological yield (kg ha^–1^)				
Normal irrigation	8513d	9209c	10313b	10502a
Drought stress at tillering stage	7113h	7794g	8445f	8542e
Drought stress at anthesis stage	6735l	7246k	8024j	8316i
Harvest index				
Normal irrigation	43.5a	41.9b	38.6h	38.8g
Drought stress at tillering stage	41.5c	40.5d	39.2f	39.3e
Drought stress at anthesis stage	36.5j	36.6i	35.2k	35.1l

*Means with various letters are significantly different according to the least significant difference (LSD) test at 0.05 probability level.

**Table 2:** Analysis of variance (ANOVA) of different parameters affected by different treatments under different drought stress conditions.

Variable	Drought	Treatment	Drought × treatment
Degree of freedom	2	3	6
Plant height	***	***	***
Spike length	***	***	***
No. of fertile tillers m^–2^	***	***	***
No. of spikelets spike^–1^	***	***	***
No. of grain spike^–1^	***	***	NS
1000 grain weight	***	***	NS
Grain yield	***	***	***
Biological yield	***	***	***
Harvest index	***	***	***
Leaf area index	***	***	***
Relative water contents	***	***	***
Stomatal conductance	***	***	***
Chlorophyll contents	***	***	***
Nitrogen contents in grains	***	***	NS
Phosphorus contents in grains	***	***	***
Potassium contents in grains	***	***	NS
Ascorbate peroxidase	***	***	NS
Catalase	***	***	***
Peroxidase	***	***	**
Superoxide dismutase	***	***	***

where *NS* non-significant at *p* ≤ 0.05, ** = significant at *p* ≤ 0.01 and *** = significant at* p* ≤ 0.001.

A significant difference in spike length was recorded under different treatments and drought stress levels (Table 1). Spikes with more length were recorded in D_0_ (17.08) and spikes with minimum length were reported in D_1_ (11.17). Plots receiving T_3_ (NBC + BR) resulted in the maximum spike length of (17.08). Statistically significant (*p* ≤ 0.001) interactive effect of both factors was reported on wheat spike length (Table 2).

A significant difference in the number of fertile tillers was recorded according to treatments and drought stress levels (Table 1). The highest number of tillers were recorded in D_0_ (410) as BR or NBC + BR were applied and minimum at D_1_ (310) at the control treatment (T_0_). The maximum number of fertile tillers was recorded in plots receiving T_3_ (NBC + BR). Statistically non-significant (*p* ≤ 0.05) interactive effect of both factors was reported on wheat tillers (Table 2).

Both treatment and drought stress levels had a significant impact on the number of spikelets spike^–1^. The maximum number of spikelets spike^–1^ (19.06) was recorded with the application of T_3_ under D_0_ while that of the minimum (13.67) with T_0_ under D_2_. Graph represented that T_3_ (NBC + BR) resulted in the maximum number of spikelets spike^–1^. Statistically significant (*p* ≤ 0.001) interactive effect of both factors was reported on wheat spikelets (Table 2).

The number of grains spike^–1^ was significantly controlled by both factors i.e. treatments and drought stress levels**.** The maximum number of grains spike^–1^ were recorded in D_0_ (38) @ T_3_ and the minimum as drought stress was applied at the anthesis stage i.e. D_2_ (27). However, this was mitigated by the combined application of NBC and BR which resulted in a 22% recovery (33).

Similarly, drought stress at tillering stage resulted in 29.2 grains spike^–1^ but NBC and BR applications showed a promising increase of 6% and 13% respectively while the T_3_ (NBC + BR) recovered 16%. Non-significant interaction was reported between treatment and drought stress (Table 2).

A significant difference in 1000 grain weight was recorded according to treatments and drought stress levels. 1000 grains with more weight were recorded in D_0_ (37) and the minimum was reported at D_2_ (25.5) @ control treatment (T_0_). Plots receiving T_3_ (NBC + BR) resulted in the maximum weight of grain. Non-significant interactive effect of both factors was reported on wheat 1000 grain weight (Table 2).

A significant difference in grain yield was recorded according to treatments and drought stress levels. Plot having control treatment showed maximum grain yield at D_0_ (3707 kg ha^–1^) but as drought stress was applied this value reduced to 2949 kg ha^–1^ (20%) and 2457 kg ha^–1^ (33%) at D_1_ and D_2_ respectively. Promising responses of 8% and 12% recovery were observed after T_1_ and T_2_ application at D_1_. Similarly, at D_2_, 8% and 15% gain was seen after NBC and BR incorporation. However, an 18% loss could be reduced as NBC and BR co-applied (T_3_). A significant interactive effect was found between both factors with maximum grain yield (Table 2).

A significant difference in biological yield was recorded according to treatments and drought stress levels. The biological yield was maximum at D_0_ (10,502 kg ha^–1^) and minimum reported at D_2_ (6735 kg ha^–1^). Plot received T_3_ (NBC + BR) reduced the 20% drought stress effect at the tillering stage and 23% at anthesis stage. A significant interactive effect was found between both factors with maximum biological yield (Table 2).

A significant difference has been observed in the harvest index according to treatments and drought stress levels. Drought stress at tillering stage caused a decrease in the harvest index from 43.5 to 41.5 while drought stress at anthesis stage dipped to 36.5. The maximum HI values were recorded in plots receiving T_3_ (NBC + BR). A statistically significant interactive effect of both factors was reported on the wheat harvest index (Table 2).

### Physiological and biochemical attributes

**Figure 1:**
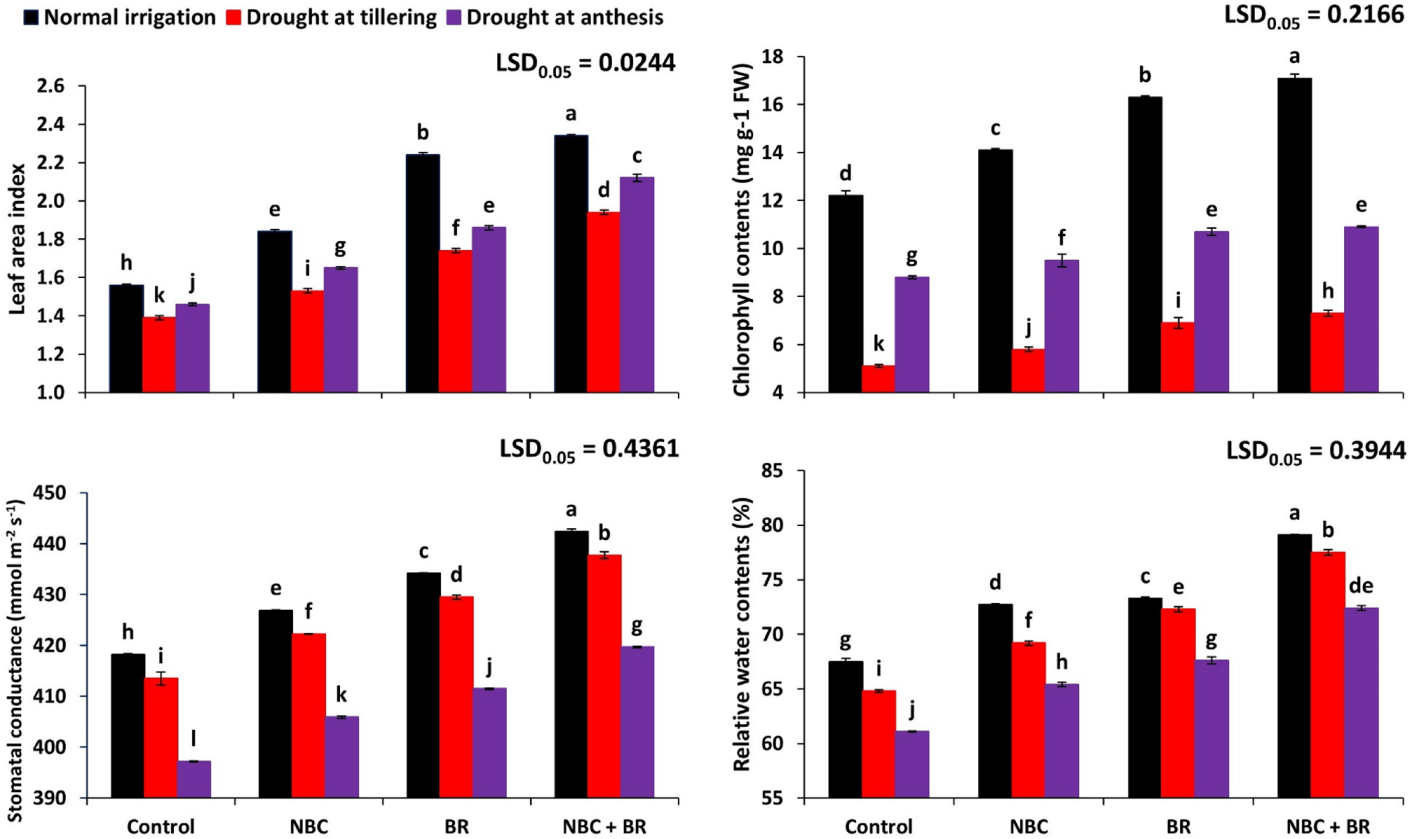
Effect of nano-biochar (NBC) and brassinosteroids (BR) on wheat leaf area index, chlorophyll contents, relative water contents, and stomatal conductance under drought stress. Bars with different letters are significantly different according to the least significant difference (LSD) test at a 0.05 probability level.

A significant difference has been observed in leaf area index (LAI) according to treatments and drought stress levels (Fig. 1). Drought stress at tillering stage significantly decreased LAI from 1.56 to 1.39 while at anthesis stage, it decreased up to 1.46. Plots receiving T_3_ (NBC + BR) resulted in the maximum LAI (2.34) of wheat plants. Statistically significant (*p* ≤ 0.001) interactive effect of both factors was reported on wheat LAI (Table 2). A significant difference has been observed in stomatal conductance according to treatments and drought stress levels (Fig. 1). Drought stress at tillering stage resulted in the minimum stomatal conductance (397.2). The combined application of NBC + BR resulted in the maximum stomatal conductance (442.4) under normal conditions while it was 419.7 under drought stress at the anthesis stage. Statistically significant (*p* ≤ 0.001) interactive effect of both factors was reported on wheat stomatal conductance (Table 2).

A significant difference has been observed in chlorophyll contents according to treatments and drought stress levels (Fig. 1). Drought stress at tillering stage caused a significant decrease in chlorophyll contents from 12.2 to 5.1 while drought stress at anthesis stage decreased it to 8.8. Plots receiving T_3_ (NBC + BR) resulted in the maximum chlorophyll contents (16.3) under normal conditions. Statistically significant (*p* ≤ 0.001) interactive effect of both factors was reported on wheat chlorophyll contents (Table 2). A significant difference has been observed in relative water contents according to treatments and drought stress levels (Fig. 1). A similar decrease with drought stress at tillering and anthesis stages. The maximum relative water contents (79.1) were recorded in plots receiving T_3_ (NBC + BR). Statistically significant (*p* ≤ 0.001) interactive effect of both factors was reported on wheat relative water contents (Table 2).

**Figure 2:**
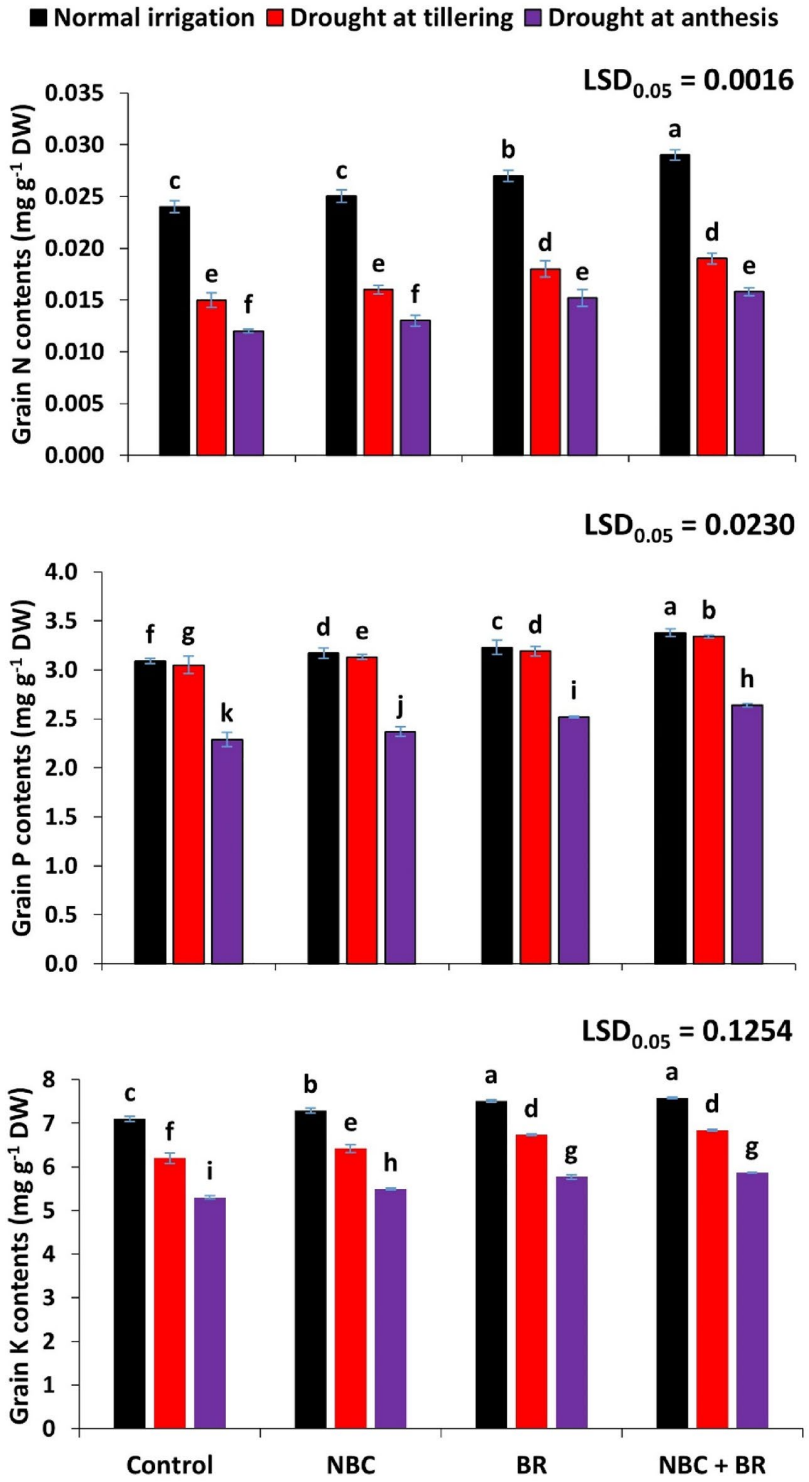
Effect of nano-biochar (NBC) and brassinosteroids (BR) wheat grain nitrogen (N), phosphorous (P), and potassium (K) contents under drought stress. Bars with different letters are significantly different according to the least significant difference (LSD) test at a 0.05 probability level.

Drought stress and treatments had a significant effect on the nitrogen contents of wheat grain (Fig. 2). Statistically maximum N contents were reported at D_0_ (0.029 mg g^*–*1^) at T_3_ (NBC + BR) and the lowest contents were recorded in D_2_ (0.012) at T_0_. Drought stress at the anthesis stage caused a 50% reduction in nitrogen uptake which was recovered (31%) at co-application of NBC + BR (0.026).

Phosphorous content showed a significant effect at drought stress and different amendments (Fig. 2). Statistically maximum P contents were reported at D_0_ (3.38) at T3 (NBC + BR) and the lowest contents were recorded in D_2_ (2.29 mg g^–1^) at T_0_ (control). Drought stress caused a 25% reduction in P content at D_2_ which was mitigated through NBC and BR application. Results showed that 15% P content was recovered at D_2_ as NBC and BR co-applied (T_3_).

Results regarding K accumulation in grains indicated that enhanced accumulation occurred in the control treatment of T_1_, T_2_ and T_3_ (Fig. 2). Drought stress at tillering stage caused a 12% loss while drought stress at anthesis stage caused a 25%. These losses were seen mitigated by 10% at D_1_ and D_2_ after the co-application of NBC + BR. Drought stress and treatments had a significant effect on the potassium contents of wheat grain.

Statistically significant (*p* ≤ 0.001) interactive effect of both factors was reported on phosphorus contents in grains while it was statistically non-significant (*p* ≤ 0.05) in case of the both nitrogen and potassium contents in grains of wheat (Table 2).

### Antioxidant response

**Figure 3:**
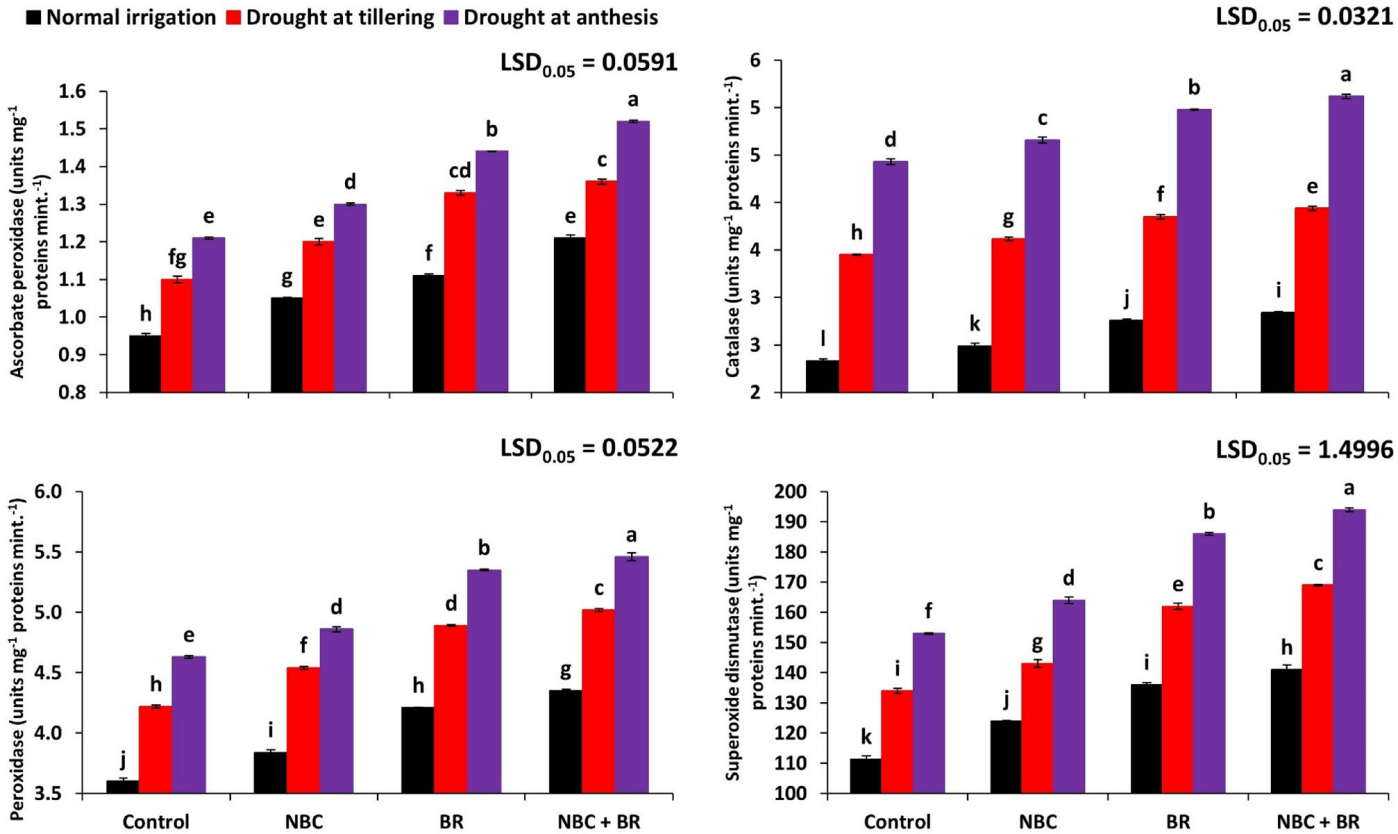
Effect of nano-biochar (NBC) and brassinosteroids (BR) on ascorbate peroxidase, catalase, peroxidase, and superoxide dismutase activities of Wheat under drought stress. Bars with different letters are significantly different according to the least significant difference (LSD) test at a 0.05 probability level.

Statistical analysis of ascorbate peroxidase showed that APX activity was significantly controlled by various treatments and drought stress levels during the study (Fig. 3). The maximum rate of APX activity was recorded in D_2_ at treatment T_3_ (1.52) and the minimum in D_0_ (0.95) at T_0_. The regression graph of drought stress showed the coefficient of regression 97% and 96% at D_1_ and D_2_ which indicated the reliability of the study at the field level. Catalase, peroxidase, and superoxide dismutase activities were significantly decreased under drought stress at tillering and anthesis stages (Fig. 3). Maximum CAT, POD, and SOD were recorded at T_3_ when NBC and BR were co-applied. We recorded 15, 17, and 26% increases in CAT, POD, and SOD respectively under the co-application of NBC and BR at D_2_ as compared to their control treatments. Similar responses were also recorded at D_1_ as 14%, 19%, and 26% improvements were seen in CAT, POD, and SOD in respective to the control. Interestingly, the same trend was seen during the control treatment after the application of NBC and BR. The regression coefficient of above 94% in CAT, POD, and SOD showed the reliability of the experiment as well. In the case of the interactive effect of both factors, a statistically significant (*p* ≤ 0.001) interaction was noted in the case of catalase, peroxidase, and superoxide dismutase while it was statistically non-significant (*p* ≤ 0.05) in case of the ascorbate peroxidase of wheat (Table 2).

### Pearson correlation

Pearson correlation was calculated among different growth, physiological, biochemical, and antioxidant activities of wheat under different treatments and drought stress levels (Table 3). Generally, the different growth, yield, physiological, and biochemical attributes of wheat plants were significantly and positively correlated with each other (Table 3). Growth parameters such as plant height, had a negative but significant correlation with ascorbate peroxidase, catalase, peroxidase, and superoxide dismutase while spike length has non-significant relation with ascorbate peroxidase, catalase, peroxidase, and superoxide dismutase. Biochemical parameters such as nitrogen, phosphorus, and potassium contents in grains samples of wheat had a negative but significant correlation with ascorbate peroxidase, catalase, peroxidase, and superoxide dismutase. The biological yield had a negative and non-significant correlation with ascorbate peroxidase, peroxidase, and superoxide dismutase but a significant and positive correlation with catalase. Grain yield, harvest index, and 1000 grain weight had a negative but significant correlation with ascorbate peroxidase, catalase, peroxidase, and superoxide dismutase.

**Table 3:** Pearson correlation among different parameters.

	AOX	BY	CAT	CC	TGW	GY	HI	K	LAI	N	NFT	NGS	NSPS	P	PH	POD	RWC	SC	SL
BY	− 0.1824 ns																		
CAT	0.8628***	− 0.5875***																	
CC	− 0.2438 ns	0.8096***	− 0.4503**																
TGW	− 0.5801***	0.7640***	− 0.8948***	0.4107*															
GY	− 0.4958**	0.9142***	− 0.8468***	0.6750***	0.9399***														
HI	− 0.8214***	0.1839 ns	− 0.8548***	− 0.0271 ns	0.7356***	0.5650***													
K	− 0.5000**	0.8749***	− 0.8533***	0.5704***	0.9702***	0.9854***	0.6154***												
LAI	0.3002 ns	0.8488***	− 0.0989 ns	0.7419***	0.3552*	0.5787***	− 0.3169 ns	0.5217**											
N	− 0.4715**	0.9281***	− 0.8071***	0.7894***	0.8651***	0.9709***	0.4645**	0.9315***	0.6363***										
NFT	− 0.4661**	0.8907***	− 0.7474***	0.8819***	0.7389***	0.9092***	0.3802*	0.8474***	0.6308***	0.9482***									
NGS	− 0.0523 ns	0.9333***	− 0.4926**	0.6665***	0.7273***	0.8537***	0.1808 ns	0.8375***	0.8221***	0.8282***	0.7916***								
NSPS	− 0.2655 ns	0.9044***	− 0.6689***	0.6218***	0.8473***	0.9287***	0.4137*	0.9091***	0.6752***	0.8867***	0.8081***	0.8814***							
P	− 0.3795*	0.6884***	− 0.7417***	0.2078 ns	0.9475***	0.8461***	0.6708***	0.9049***	0.3564*	0.7365***	0.5605***	0.7162***	0.8193***						
PH	− 0.5675***	0.8731***	− 0.8409***	0.7489***	0.8592***	0.9365***	0.4962**	0.9033***	0.5609***	0.9527***	0.8830***	0.7559***	0.8564***	0.7267***					
POD	0.9718***	− 0.2872 ns	0.9202***	− 0.3334*	− 0.6595***	− 0.5958***	− 0.8504***	− 0.5875***	0.1999 ns	− 0.5844***	− 0.5705***	− 0.1642 ns	− 0.3760*	− 0.4541**	− 0.6530***				
RWC	0.1616 ns	0.8304***	− 0.3216 ns	0.4560**	0.6512***	0.7340***	0.0987 ns	0.7392***	0.8136***	0.6949***	0.5877***	0.8653***	0.8536***	0.7442***	0.6146***	0.0468 ns			
SC	− 0.0553 ns	0.8548***	− 0.5181**	0.4108*	0.8224***	0.8396***	0.3114 ns	0.8672***	0.7056***	0.7704***	0.6284***	0.8791***	0.8954***	0.8953***	0.7185***	− 0.1520 ns	0.9545***		
SL	0.2338 ns	0.7752***	− 0.0931 ns	0.8321***	0.2466 ns	0.5270**	− 0.3036 ns	0.4408**	0.9102***	0.6224***	0.6932***	0.7320***	0.6076***	0.1831 ns	0.5107**	0.1302 ns	0.6836***	0.5380***	
SOD	0.9720***	− 0.2703 ns	0.9231***	− 0.2726 ns	− 0.6790***	− 0.5905***	− 0.8720***	− 0.5937***	0.2315 ns	− 0.5633***	− 0.5270**	− 0.1588 ns	− 0.3729*	− 0.4938**	− 0.6435***	0.9900***	0.0387 ns	− 0.1763 ns	0.179 ns

where *AOX* Ascorbate peroxidase; *BY* Biological yield; *CAT* Catalase; *CC* = Chlorophyll contents; *TGW* 1000 grain weight; *GY* Grain yield; *HI* Harvest index; *K* Potassium contents in grains; *LAI* Leaf area index; *N* Nitrogen contents in grains; *NFT* No. of fertile tillers; *NGS* No. of grains spike^–1^; *NSPS* No. of spikelets spike^-1^; *P* Phosphorus contents in grains; *PH* Plant height; *POD* Peroxidase; *RWC* Relative water contents; *SC* Stomatal conductance; *SL* Spike length; *SOD* Superoxide dismutase; *NS* non-significant at *p* ≤ 0.05, * = significant at *p* ≤ 0.05, ** = significant at *p* ≤ 0.01 and *** = significant at* p* ≤ 0.001.

## Discussion

Water scarcity affects plant height and growth negatively^54,55^. Under drought stress plant height can be increased by supplying such soil fixation and growth regulating agents that benefit both crop and soil physical and chemical health. Maximum plant height was achieved by adding nano-biochar (NBC) and brassinosteroids (BR) under normal irrigation, the increase in plant height was due to the positive influence of both agents. This study is supported by Raza et al.^56^ that biochar and plant growth-promoting regulators help in promoting plant height.

Spike length plays a vital role in determining half of the yield-determining attributes greater the spike length more will be the crop yield ultimately as increased spike length produces an increased number of spikelets spike^–1^ which promotes higher grain formation. Like other growth and development stages of crop water availability affects spike length and to attain maximum spike length crop yield and growth-enhancing amendments are required with normal irrigation. Drought stress had a serious negative relation with wheat spike length. For eliminating the negative impact of drought stress, BR and NBC treatments were tested which showed an increase in spike length by treatment having both NBC and BR. This study is supported by the statements of Almeselmani et al.^57^ where a 16.61% increase in spike length was observed.

The number of fertile tillers determines the crop yield. Grain production and count increase with the increase in fertile tillers population. Drought stress at any stage of crop growth and development restricts the tiller fertility thus lowering grain count and weight. An increase in fertile tillers was recorded as the result OF NBC + BR under control irrigation. According to Ramraj et al*.*^58^ exogenous applications of BR increase the number and degree of fertile tillers and spikes respectively whereas^59^ biochar increases crop growth and yield attributes.

The number of spikelets spike^–1^, the number of grains spike^–1^, and 1000 grain weight are directly related to crop yield. Drought stress causes a reduction in all these attributes thus producing low yield. The number of spikelets spike^–1^ is reduced under drought stress due to the death of floret sets at the terminal and basal ends whereas the number of grains spike^–1^ was lowered due to the dehydration of the pollen grains^60^. 1000 grain weight was also determined significantly by drought stress as the maximum 1000 grain weight was obtained under normal irrigation as floret sets and pollen grain development was boosted which led to a higher 1000 grain weight. NBC + BR application resulted in a higher number of grains spike^–1^, 1000 grain weight, and the number of spikelets spike^–1^. According to Wang et al.^59^, biochar application increases the number of spikelets spike^–1^ in wheat, the number of grains, and 1000 grain weight in rice and wheat respectively.

The final aim of crop production is to gain maximum grain yield. The grain yield of the crop depends upon several yield attributes and unfortunately drought stress harmed those yield attributes. The occurrence of drought stress at critical growth stages is harmful as reported by Raza et al.^61^. Drought stress at anthesis stage causes maximum loss. In this study, an increase in grain yield was reported by NBC + BR application under no drought stress. The biological yield represents the dry accumulation by the crop during the entire season. Biological yield and drought stress relation are reported as same as others BY increases with decrease or elimination of drought stress and vice versa.

The crop plant portioning ability of photosynthates towards economical parts is determined by Harvest Index. An increase in the harvest index reflects an improvement in crop growth and development. The lowest harvest index was reported under drought stress at anthesis stages as it lowered grain production and yield whereas NBC and BR application combined under normal irrigation resulted in an increased harvest index. Improvement in grain yield to biomass (HI) due to the improved plant biomass as the result of BR application was stated by Hnilicka et al.^62^.

Among plant growth and development-promoting nutrients^63^, nitrogen is the most important and commonly used and required nutrient. Phosphorous and Potassium are also among other nutrients required by plants regularly^64–66^. Under drought stress, potassium is required by the plants for maintaining the turgidity and osmotic potential whereas under low moisture uptake of P and K is restricted. Combined application of NBC and BR significantly increased NPK contents of grains in wheat. This is because biochar increased organic matter in the soil and improved water retention of sandy loam soil^50^ which leads to an increase in NPK uptake.

From the results stated above, it is obvious that water stress increased the secretion of ROS in wheat. This overproduction might be to mitigate the prevailing drought stress as stated by^56,57^. At D_1_ and D_2_, APX, CAT, POD, and SOD production was enhanced compared to respective control treatments. Biochar concentrations increased the antioxidant activities in the wheat plants by improving cell growth, and soil–plant water relationship^67–69^. Nanoparticles increased the POD and APX activity to mitigate the water scarcity situation as reported by^70–73^. Correlation analysis showed a linear relationship among treatments and recommended the usage of brassinosteroids for increasing stomatal conductance, leaf area index, relative water contents, and chlorophyll contents and for ameliorating the effect of drought stress.

## Conclusions

The results showed that the combined application of brassinosteroids (BR) and nano-biochar (NBC) had an ameliorating impact against drought stress and a synergistic impact on the growth, yield, physiological, and biochemical attributes of wheat. Drought stress significantly reduced the growth, yield, physiological, and biochemical attributes of wheat. This stress was ameliorated with the application of BR and NBC alone or combined. The combined application of BR and NBC had a significant and synergistic impact on growth (plant height, spike length, and no. of spikelets spike^–1^), yield (no. of fertile tillers m^–2^, grain yield, biological yield, harvest index), physiological (leaf area index, relative water contents, stomatal conductance, and chlorophyll contents) and biochemical attributes (catalase, peroxidase, superoxide dismutase, and phosphorus contents in grains) while non-significant with no. of grains spike^–1^, 1000 grain weight, nitrogen, and potassium contents in grains and ascorbate peroxidase. In conclusion, the combined application of BR and NBC could ameliorate the negative impacts of drought on growth, yield, physiological, and biochemical attributes of wheat under field conditions. To authenticate the efficacy of tested amendments, more field and laboratory trials involving different crops under different climatic conditions are needed in the future.

## Data Availability

All data generated or analyzed during this study are included in this submitted article.
